# Intrinsic Altruism or Social Motivation—What Does Pupil Dilation Tell Us about Children's Helping Behavior?

**DOI:** 10.3389/fpsyg.2017.02089

**Published:** 2017-12-05

**Authors:** Carolina Pletti, Anne Scheel, Markus Paulus

**Affiliations:** ^1^Developmental Psychology Unit, Department of Psychology, Ludwig Maximilian University of Munich, Munich, Germany; ^2^School of Innovation Sciences, Eindhoven University of Technology, Eindhoven, Netherlands

**Keywords:** prosocial, pupillometry, helping, toddlers, altruism

Experimental and observational evidence shows that children, from around 18 months of age onwards, perform actions that can be interpreted as “helping.” For instance, they hand back fallen objects or want to participate in household activities (e.g., Rheingold, 1982; Warneken and Tomasello, 2006; Brownell, 2011; Carpendale et al., 2015). A growing amount of research is trying to understand the psychological basis behind this behavior (for reviews see Paulus, 2014; Brownell, 2016; see also Warneken, 2015, for an evolutionary perspective). For example, is young children's “helping” due to an interest in the wellbeing of others? Or does it reflect a motivation to interact with other people and to be involved in their actions? In order to understand what drives children's helping, a number of recent studies have used pupil dilation as a measure of children's arousal (Hepach et al., 2012, 2016a,b), concluding that children's motivation to help is intrinsically altruistic. This opinion piece aims at re-examining those findings, suggesting that they are also compatible with alternative explanations.

In the studies discussed here, pupil diameter is measured by means of an eye tracker. Changes in pupil diameter reflect the contraction or dilation of the sphincter and the dilator muscles, innervated (respectively) by the parasympathetic and sympathetic branches of the autonomic nervous system. Changes in pupil dilation (when light conditions are kept constant) thus reflect changes in autonomic arousal (Beatty and Lucero-Wagoner, 2000). The advantage of using pupillometry is that it provides information which cannot be accessed with behavioral measures and a quantitative measure of the psychological processes underlying a certain behavior (Hepach and Westermann, 2016). One drawback of pupil dilation is that it is an unspecific measure of autonomic activation, which might result from a variety of cognitive and emotional processes (Beatty and Lucero-Wagoner, 2000; Sirois and Brisson, 2014). This ambiguity does not (without tight control conditions) allow to infer which psychological process is causing the arousal. Despite this fact, pupil dilation assessments are potentially useful in developmental research: They give a quantitative measure of the psychological processes related to a certain event which is not influenced by shyness or verbal fluency because children are not required to provide an overt response (Hepach and Westermann, 2016). Hepach et al. (2012, 2016a,b) for the first time applied this measure in the context of instrumental helping to assess children's autonomic arousal when perceiving a situation in which someone displays a need for help.

## The prosocial arousal hypothesis

In a series of experiments, pupil dilation was used to measure 2-year-old children's arousal state while observing an adult needing help (for instance, drawing a picture and dropping the crayon they need, or stacking objects and dropping the last one needed to complete a pile). Then, children were either given the opportunity to help or observe someone else helping (Hepach et al., 2012, 2016a,b).

According to the authors, these studies show that:

1 - 2-year-olds show increased arousal after seeing an adult in need of help
2 - The degree of arousal predicts the speed with which children help the adult
3 - The arousal diminishes after the adult has received help, either by the child or by another person, but stays high if the adult does not receive help.

According to a recent review, these results indicate that toddlers have an intrinsic prosocial motivation (Hepach, 2017). The author also considers several alternative explanations for children's helping behavior: social motivation (children might not be altruistically motivated to help, but simply want to interact with the adult); goal contagion (children want to see the action completed because the adult's goal becomes their own); restoration of order (children are motivated to put back displaced objects). All of these alternatives are rejected in favor of the explanation that children are intrinsically motivated to see others helped because they genuinely care for their well-being. The author argues that “*children's motivation to help others is not only intrinsic but also inherently prosocial*” (p. 53), since the arousal “*is the physiological manifestation of children's emotional involvement and the degree to which they occupy themselves with others' unfulfilled needs*” (p. 51).

This conclusion is surprising, because the experiments discussed in the review do not convincingly refute all of the alternative explanations listed above (social motivation, goal contagion, and restoration of order). The author's argument thus relies on the assumption that the arousal measured via pupil dilation has to be prosocial and would thus only arise from other-oriented, altruistic motivation: “*When young children appraise situations in terms of another individual needing help, the increase in elicited internal arousal reflects the measurable degree of their intrinsic motivation to help*” (p. 52). Yet, given the unspecific nature of physiological arousal, which is granted by the author just a few sentences later—“(c)*hanges in pupil size do not appear to indicate the stimulus' valence*” (p. 52)—, using pupil dilation as a direct measure of prosocial motivation is not warranted: Current literature suggests that increase in pupil dilation might indicate increased attention, emotional arousal, cognitive effort such as memory processes, target detection and/or surprise (Bradley et al., 2008; Privitera et al., 2010; Preuschoff, 2011; Laeng et al., 2012; Sirois and Brisson, 2014; Verschoor et al., 2015). These processes are not systematically ruled out in the studies that the review mentions, so assuming that an increase in pupil dilation signals an increase in motivation is a big and hasty step.

## *Pro-*social or social?

In lieu of evidence validating pupil dilation as a specific measure of *prosocial arousal*, it is important to consider which predictions alternative explanations make with regard to arousal in the studies presented in Hepach (2017). In the following we take a closer look at three key studies.

First, in one study, 2-year-olds observed an adult drawing a picture and accidentally dropping the crayon from the table (or stacking cans to form a tower and dropping the last item). One group was given the opportunity to help the adult; one group was brought in front of the adult, held back, and saw that the adult was not helped; one group was brought in front of the adult, held back, and observed another person helping. At this point, children who saw that the adult was not helped showed greater pupil dilation than children in the other two groups (Hepach et al., 2012).

This result fits the *prosocial arousal* hypothesis, because an intrinsic altruistic motivation should lead to higher arousal when observing someone in need as opposed to observing the situation being resolved or resolving it oneself. However, it also fits all of the alternative hypotheses: We would expect high arousal when children are faced with an opportunity to join in a contingent interaction (social motivation), to fulfill an agent's goal (goal contagion), or to tie up loose ends (restoration of order), compared to when they can act on their respective motivation or when the opportunity has passed because the situation was resolved by a third party. Furthermore, the greater pupil dilation in the no-help condition could reflect a greater memory load due to keeping in mind the unresolved action.

Second, in another experiment, 2-year-olds observed a scene in which an adult needed help to complete an action because they dropped an object (Hepach et al., 2016a). Children were then given the opportunity to help by picking up the object. Here, the degree of pupil dilation predicted the speed with which the children picked up an object, yet—importantly—not necessarily the needed object. There were 12 objects scattered on the floor, only half of which pertained to the experimenter's activity. There was no difference in pupil dilation between children who picked up a relevant or an irrelevant object first, and pupil dilation predicted action latency independently of object relevance. Moreover, children kept picking up objects after having picked up all relevant ones. Can we still say that they were helping?

Here, the results actually favor the social motivation hypothesis: If children's arousal reflected the motivation to fulfill the experimenter's need, we would expect it (a) to be higher in children that seek out relevant objects first and (b) to predict action latency only for children that pick up relevant objects. Whereas if it would reflect the motivation to engage in a contingent interaction with the experimenter—picking up objects as part of an interactive game—we would expect exactly the pattern of results that emerged in the study. Thus, this study actually provides evidence for the claim that young children's helping behavior is driven by a motivation to interact with others (Paulus, 2014).

Finally, in a third study, 2- and 3-year olds were led to believe that they damaged the experimenter's playground by spilling water on it. Then they got the opportunity to help by handing them a towel. In one condition, they successfully completed the action. In another condition, just as they reached for the towel, another adult took it and handed it to the experimenter. Results showed that children who could not repair the damage showed greater pupil dilation than children who could (Hepach et al., 2016b).

In our opinion, this result represents direct evidence *against* the prosocial arousal hypothesis: While the authors argue that the increased arousal of children who could not repair the damage themselves can be seen as a sign of guilt, which itself would arise from an intrinsic prosocial motivation, this pattern would in fact *not* be expected for a truly intrinsic, other-oriented motivation. If children were only concerned with the experimenter's well-being, their arousal should recede regardless of who provided the help (as actually shown in Hepach et al., 2012). Thus, the result rather speaks for an extrinsic, self-oriented motivation, in which children's arousal is a function of expected (social) consequences for themselves. Moreover, the results can be explained equally well by the social motivation hypothesis: children who could not successfully comply with the experimenter's first request (spilling the water instead of handing it over), may have been particularly frustrated when they could not even perform the second one (handing a towel), which causes increased pupil dilation. Interestingly, most children did not attempt to clean up themselves, but handed the towel to the experimenter instead. This might be an indication that children wanted to participate in a joint task, rather than help by repairing the damage.

To summarize, the pattern of results seems to actually contradict the claim of an intrinsic and altruistic prosocial motivation. Moreover, none of the studies described above allows to rule out the social motivation hypothesis in favor of the prosocial arousal hypothesis.

## Additional concerns and conclusions

In addition, it should be noted that each of the studies reported in the review by Hepach (2017) uses new manipulations and analyses, meaning that each single result is never directly replicated. For instance, an increase in arousal from baseline after seeing a person needing help compared to seeing displaced objects is reported only in Experiment 2 of Hepach et al. (2016a), and a correlation between arousal and helping speed only in Experiment 1 of the same paper. It would be desirable to replicate these findings before drawing strong theoretical conclusions^1^.

Although prosocial arousal is an intriguing hypothesis, we argue that the results presented in these studies do not warrant strong theoretical conclusions. In particular, they fail to exclude the social motivation hypothesis. This hypothesis is especially compelling if one considers that from early infancy, children are gradually and persistently encouraged to participate in collaborative activities with adults (Rheingold, 1982; Carpendale et al., 2013; Hammond, 2014; Dahl, 2015). Thus, children might be motivated to help because they are used to—and enjoy—taking part in joint tasks with adults. In this view, not only the social interaction in itself would be rewarding, but also the ability to comply with the experimenter's more or less explicit requests (Rheingold et al., 1987).

A second concern is that in most of the studies discussed by Hepach (2017)—as is the case in other studies (e.g., Svetlova et al., 2010)—children received cues from the experimenter, which increased over time. Usually the experimenter would first look at the object they needed, then verbalize their need, and finally explicitly ask children to give them the object. In these studies, only a few children reacted immediately. Most of them required several cues before acting, and some did not help at all. This means that children's helping behavior was elicited rather than spontaneous, which speaks for extrinsic rather than intrinsic motivation.

To conclude, this line of research provides a fruitful and intellectually stimulating contribution to the study of early helping. The work of Hepach and colleagues shows that children pay attention to social scenes and are sensitive to others' requests, which in itself is important information. However, we cannot state that pupil dilation in these studies is due to a genuinely altruistic motivation, rather than a desire to interact with the experimenter, or to comply with her requests. To distinguish between these interpretations, one could add further control conditions, e.g., a condition in which the child has the opportunity to interact without helping, or a condition in which no requests are made to the child. In any case, pupil dilation does not inform us about which particular process is underlying the arousal, which could be related not only to a concern for others, but also to different memory demands, children's excitement to interact with others, or self-referential emotions such as guilt. This should be kept in mind by future studies. Thus, the debate on what motivates children's instrumental helping is still ongoing (Paulus, 2018).

## Author contributions

All authors listed have made a substantial, direct and intellectual contribution to the work, and approved it for publication.

### Conflict of interest statement

The authors declare that the research was conducted in the absence of any commercial or financial relationships that could be construed as a potential conflict of interest. The reviewer CB and handling Editor declared their shared affiliation.

## Footnotes

^1^A step in this direction has been recently made in Hepach et al., (2017), where a joint re-analysis of data from several studies shows again that pupil dilation predicts acting speed in instrumental helping tasks.

## References

[B1] Beatty J. Lucero-Wagoner B. (2000). The pupillary system, in Handbook of Psychophysiology, eds Cacioppo J. T. Tassinary L. G. Berntson G. G. (New York, NY: Cambridge University Press), 142–162.

[B2] Bradley M. M. Miccoli L. Escrig M. A. Lang P. J. (2008). The pupil as a measure of emotional arousal and autonomic activation. Psychophysiology 45, 602–607. 10.1111/j.1469-8986.2008.00654.x 18282202 PMC3612940

[B3] Brownell C. A. (2011). Early developments in joint action. Rev. Philos. Psychol. 2, 193–211. 10.1007/s13164-011-0056-1 23087769 PMC3474705

[B4] Brownell C. A. (2016). Prosocial behavior in infancy: the role of socialization. Child Dev. Perspect. 10, 222–227. 10.1111/cdep.12189

[B5] Carpendale J. I. Hammond S. I. Atwood S. (2013). A relational developmental systems approach to moral development. Adv. Child Dev. Behav. 45, 125–153. 10.1016/B978-0-12-397946-9.00006-3 23865115

[B6] Carpendale J. I. M. Kettner V. A. Audet K. N. (2015). On the nature of toddlers' helping: helping or interest in others' activity? Soc. Dev. 24, 357–366. 10.1111/sode.12094

[B7] Dahl A. (2015). The developing social context of infant helping in two u.s. samples. Child Dev. 86, 1080–1093. 10.1111/cdev.12361 PMC4575818 25800068

[B8] Hammond S. I. (2014). Children's early helping in action: piagetian developmental theory and early prosocial behavior. Front. Psychol. 5:759. 10.3389/fpsyg.2014.00759 25101027 PMC4102168

[B9] Hepach R. (2017). Prosocial arousal in children. Child Dev. Perspect. 11, 50–55. 10.1111/cdep.12209

[B10] Hepach R. Vaish A. Grossmann T. Tomasello M. (2016a). Young children want to see others get the help they need. Child Dev. 87, 1703–1714. 10.1111/cdev.12633 28262942

[B10a] Hepach R. Vaish A. Müller K. Tomasello M. (2017). The relation between young children's physiological arousal and their motivation to help others. Neuropsychologia. [Epub ahead of print]. 10.1016/j.neuropsychologia.2017.10.010 29030228

[B11] Hepach R. Vaish A. Tomasello M. (2012). Young children are intrinsically motivated to see others helped. Psychol. Sci. 23, 967–972. 10.1177/0956797612440571 22851443

[B12] Hepach R. Vaish A. Tomasello M. (2016b). Children's intrinsic motivation to provide help themselves after accidentally harming others. Child Dev. 88, 1251–1264. 10.1111/cdev.12646 27800601

[B13] Hepach R. Westermann G. (2016). Pupillometry in infancy research. J. Cogn. Dev. 17, 359–377. 10.1080/15248372.2015.1135801

[B14] Laeng B. Sirois S. Gredebäck G. (2012). Pupillometry: a window to the preconscious? Perspect. Psychol. Sci. 7, 18–27. 10.1177/1745691611427305 26168419

[B15] Paulus M. (2014). The emergence of prosocial behavior: Why do infants and toddlers help, comfort, and share? Child Dev. Perspect. 8, 77–81. 10.1111/cdep.12066

[B16] Paulus M. (2018). The multidimensional nature of early prosocial behavior: a motivational perspective. Curr. Opin. Psychol. 20, 111–116. 10.1016/j.copsyc.2017.09.003 28988024

[B17] Preuschoff K. (2011). Pupil dilation signals surprise: evidence for noradrenaline's role in decision making. Front. Neurosci. 5:115. 10.3389/fnins.2011.00115 21994487 PMC3183372

[B18] Privitera C. M. Renninger L. W. Carney T. Klein S. Aguilar M. (2010). Pupil dilation during visual target detection. J. Vis. 10:3. 10.1167/10.10.3 20884468

[B19] Rheingold H. L. (1982). Little children's participation in the work of adults, a nascent prosocial behavior. Child Dev. 53:114 10.2307/1129643

[B20] Rheingold H. L. Cook K. V. Kolowitz V. (1987). Commands activate the behavior and pleasure of 2-year-old children. Dev. Psychol. 23, 146–151.

[B21] Sirois S. Brisson J. (2014). Pupillometry. Wiley Interdiscipl. Rev. Cogn. Sci. 5, 679–692. 10.1002/wcs.1323 26308873

[B22] Svetlova M. Nichols S. R. Brownell C. A. (2010). Toddlers' prosocial behavior: from instrumental to empathic to altruistic helping. Child Dev. 81, 1814–1827. 10.1111/j.1467-8624.2010.01512.x 21077866 PMC3088085

[B23] Verschoor S. A. Paulus M. Spapé M. Biro S. Hommel B. (2015). The developing cognitive substrate of sequential action control in 9- to 12-month-olds: evidence for concurrent activation models. Cognition 138, 64–78. 10.1016/j.cognition.2015.01.005 25704583

[B24] Warneken F. (2015). Precocious prosociality: Why do young children help? Child Dev. Perspect. 9, 1–6. 10.1111/cdep.12101

[B25] Warneken F. Tomasello M. (2006). Altruistic helping in human infants and young chimpanzees. Science 311, 1301–1303. 10.1126/science.1121448 16513986

